# Lunacy on the Stock Exchange

**Published:** 1863-04

**Authors:** 


### Art. XII.—LUNACY ON THE STOCK EXCHANGE.

A Joint-Stock Company for the treatment of the insane, offering a large per-centage of profit to the shareholders, is certainly a startling novelty, even in this commercial age. Joint-stock boot manufactories, joint-stock bakeries, joint-stock gas and water companies, we are all familiar with, and the element of publicity contained in the annual statement of their affairs is perhaps one of their best features; but we have yet to learn that this is an element considered necessary in the management of lunatic asylums. We may be wrong, but we must confess that, on the first blush of the thing, it does seem rather strange to find respectable names in the profession proposing a scheme for making a commercial profit out of the most terrible malady that can afflict humanity, and a profit exacted, not, as in the case of private asylums, belonging to and conducted by special physicians, for the care and anxiety imposed upon the medical proprietor, but for the benefit of a body of shareholders, who in all probability would care no more for the patients under treatment in the joint-stock asylum than they would for the stones out of a quarry, or the coals out of a mine, worked and producing a profit. If the Commissioners in Lunacy have set their faces in a determined manner against any one feature in the management of private lunatic asylums, it has been against the element of profit to be found in them, and it is well known that their aversion to license new metropolitan houses arises from this cause; but here we have the element of profit paraded in its most offensive form. Imagine, good reader, the chaffering on the Stock Exchange in these Sanatorium Association shares—the slang passing between Bulls and Bears—for stockbrokers are in the habit of calling a spade a spade, and do not mince their words. Some such a conversation as this, for instance, would possibly be heard in Capel Court:—

*Brown.* How are lunatics to-day?

*Jones.* Uncommon lively. Report of Commissioners just out; amazing increase in the lunacy average.

*Brown.* Do me 50 at $5\frac{5}{8}$ premium?

*Jones.* Can't look at 'em at that figure. Sanatorium full, and new wings building right and left.

*Brown.* Say six?

*Jones.* Six—nonsense, my boy. A member of the Government and three representatives of leading constituencies just sent in—best paying speculation of the day, &c. &c.

But to leave badinage. Let us ask Dr. Stevens*—for we regret to say that he is the only member of the staff advertised in the prospectus who has any pretensions to a knowledge of the treatment of lunatics—if he can reconcile the principle on which a joint-stock association, holding out a premium of large profit to the shareholders, must act, with the latest and most enlarged views of the best method of treating mental disease? We know that all enlightened alien physicians have long come to the conclusion that to associate large numbers of the insane together is a great mistake, and that asylums of a moderate size hold out the best prospect of cure to the inmates. We believe that this is the opinion of the Commissioners, and that they are inclined to disperse patients as much as possible

---

* We are the more astonished at seeing Dr. Stevens' name associated with this joint-stock private lunatic asylum, knowing as we do his inveterate, and, we may add, *chronic* opposition to private lunatic asylums and all connected with them. It was Dr. Stevens who had the questionable taste some years ago, at a meeting of the "*Association of Medical Officers of Asylums for the Insane,*" to propose that no physician attached to a private asylum should be eligible to membership, and this, too, in the presence of men like Drs. Conolly and Sutherland!

"*The force of folly could no further go,*"

except, in the face of such a generous, liberal, and enlightened expression of opinion (?) to aid and abet in the establishment of a private asylum on a joint-stock principle, offering to the shareholders a liberal return for money invested in this trading speculation.

among the sane element of the population rather than to coop them together in enormous establishments. They have protested over and over again against such monstrous establishments as Colney Hatch. Yet what is the proposition held out by the promoters of this new scheme? They say they have taken an old mansion, surrounded with spacious grounds, at Hendon, where they intend to erect an asylum "on a commanding scale," and that "the building proposed to be erected will be so designed as to permit of being added to as required." Surely we have here a project which is intended to be just as expansive as the pockets of the shareholders will permit. The question of science, or the limitation of numbers, is not to be consulted, but simply the commercial element of profit. If the "handsome return on the capital expended" is to be realized, we well know that it can only be done by congregating the largest possible number of lunatics together, and by keeping down the staff and working expenses to the lowest possible figure. Are we come to this? Is this monstrous Colney Hatch for private patients to be the last word of science as regards the treatment of the insane?

We perceive that a private circular is especially enclosed to medical men, stating that to them a "preference" will be given in the allotment of shares. Now of course this bait is held out to the profession with the hope of gaining its support. If we are not mistaken, however, the Commissioners will have something to say to this arrangement, inasmuch as it gives medical shareholders a direct interest in filling the house with patients, a piece of jobbing which no proprietor of a private asylum is allowed, and very justly so, to indulge in. It is quite true, that by the terms of the Lunacy Act, no shareholder in this undertaking will be able to sign a certificate for this institution, but there are many ways of directing influence to bear upon patients; and we may be sure that a medical shareholder would use that influence in favour of an establishment the success of which he had a pecuniary interest in. This is a very grave objection to this joint-stock scheme, to which we wish to call the attention of all those who may be inclined to join it with the notion that they are doing a philanthropic act.

There is also another point we may remind them of. Shareholders, however numerous, in such schemes, will have to take upon themselves the duties and responsibilities of proprietors, and those of the profession who have the unhappiness to possess establishments of this kind but too well know how onerous those duties are. Thus it may happen that an unlucky speculator who has just bought shares in the open market, in charming ignorance of the Lunacy Law, or the powers of the

Commissioners, may suddenly find himself overwhelmed with penalties. This we believe to be the law of the matter; if, however, we are wrong, and shareholders are not personally responsible, we may justly ask, what faith can the public have in such an undertaking, and what chance has it of being recognised by the Commissioners?

It is not for us to say who are the chief movers in this monstrous scheme, or to hint at the motives which have prompted them, but we cannot help remarking upon the fact that before a shilling has been subscribed, we find that the medical staff, with the exception of the resident physician, has been appointed! We may ask, Have they appointed themselves? and if not, on what principle have they been selected? We are accustomed to find a speciality treated by specialists, but with the exception of one physician, late resident physician of St. Luke's Asylum, there is not one name that is in any way associated with lunacy to be found in the list of the consulting physicians and surgeons. Surely, if a new hospital for the treatment of consumption were started, the physicians who have studied that speciality would be very much astonished to find the names of Dr. Conolly, Sir A. Morison, and Dr. Munro figuring as visiting physicians. Yet it would seem that the speciality of all others which requires a life-long apprenticeship to acquire proficiency in, is handed over to medical men, able and skilled in their own departments of science we readily admit, but who are totally innocent (without any fault of their own) of any practical acquaintance with the subject of insanity. "*Ne sutor ultra crepidam.*" We are inclined to suppose that Dr. Quain has not carefully considered the questionable nature of the appointment as far as he is concerned; if he had, we feel sure that the honourable etiquette which prevents medical men from invading each other's territory, would have prevented him from damaging his fair name by associating it with a trading scheme, not only rotten in its very foundation, but one which, in the existing feeling of the more honourable portion of the medical profession, never can be carried successfully into operation.

A proposal of this kind must, we feel assured, have a most disastrous effect upon the public mind, and tend greatly to lower the medical profession as a body in the estimation of those whose good opinion we all so much covet. In the words of Garth, it will be said:—

> "The healing Art now sickening hangs its head,
> And once a *Science*, has become a *Trade*."

If Dr. Quain and his friends were to succeed in this commercial speculation, what is to prevent there being established, immediately afterwards, a "Joint-Stock Hospital for the Cure of Consumption" or the Stone, a "Sanatorium for Diseases of the Liver," a "Company, with Limited Liability, for the Successful Treatment of Skin Diseases," or an "Institution" (offering a large division of profits to the shareholders) similar in character to the Lock Hospital, specially organized for the upper classes, guaranteeing of perfect privacy to the patients under treatment?

Shades of Pinel and Esquirol! what say you to this attempt to reduce the most exalted of all sections of medical science to the level of a stock-jobbing speculation?

It must not be supposed for a moment that we do not acknowledge the want of asylums for the reception of a class of patients who, although coming from the middle class, have not sufficient means to meet the charges of private asylums. On the contrary, we think this to be one of the great wants of the day, but it must be supplied by philanthropists, such as those who support Coton Hill Asylum, and not by mere speculators who seek to make a large profit out of the sufferings of their fellows. In conclusion, we would say to those who may be contemplating applying for shares—if such there be—would it not be well before investing money in bricks and mortar, and planting out pleasure-grounds, to ascertain in the first place, whether the Commissioners in Lunacy are inclined to license the house, when all is ready for the reception of patients—a thing which, with all due deference to the flaming character of the prospectus, and the certainty with which large returns are promised to shareholders, we may be permitted to doubt.

---

